# MOSS: multi-omic integration with sparse value decomposition

**DOI:** 10.1093/bioinformatics/btac179

**Published:** 2022-03-24

**Authors:** Agustin Gonzalez-Reymundez, Alexander Grueneberg, Guanqi Lu, Filipe Couto Alves, Gonzalo Rincon, Ana I Vazquez

**Affiliations:** Department of Epidemiology and Biostatistics, Michigan State University, East Lansing, MI 48824, USA; Department of Epidemiology and Biostatistics, Michigan State University, East Lansing, MI 48824, USA; Department of Epidemiology and Biostatistics, Michigan State University, East Lansing, MI 48824, USA; Department of Epidemiology and Biostatistics, Michigan State University, East Lansing, MI 48824, USA; Genus PLC Inc., Genome Sciences R&D, De Forest, WI 53532, USA; Department of Epidemiology and Biostatistics, Michigan State University, East Lansing, MI 48824, USA

## Abstract

**Summary:**

This article presents multi-omic integration with sparse value decomposition (MOSS), a free and open-source R package for integration and feature selection in multiple large omics datasets. This package is computationally efficient and offers biological insight through capabilities, such as cluster analysis and identification of informative omic features.

**Availability and implementation:**

https://CRAN.R-project.org/package=MOSS.

**Supplementary information:**

Supplementary information can be found at https://github.com/agugonrey/GonzalezReymundez2021.

## 1 Introduction

Omic data are characterized by many features from multiple layers of data (e.g. genome, transcriptome and proteome). Thus, traditional methods (e.g. ordinary least squares) are insufficient to obtain significant insights from this multi-layer, high-dimensional data. To effectively integrate multi-omic data, novel methods have been developed (González-Reymúndez *et al.*, 2017; Lock *et al.*, 2013; Rohart *et al.*, 2017; Shen *et al.*, 2009, 2016; Zhang *et al.*, 2016). These methods have profoundly contributed to our understanding of variation in complex traits across diverse levels of regulation (e.g. mutations in coding genes and epigenetic regulation) (Hasin *et al.*, 2017; Ritchie *et al.*, 2015).

Thanks to ongoing data collection efforts, omic data increase in the number of features and available samples. This increase in sample size provides more opportunity for inference and prediction of characteristics of interest (Müller *et al.*, 2020). However, more extensive data sizes can make computations progressively lengthier and impossible to perform in some cases (Mangul *et al.*, 2019). Moreover, extensive data sizes also compromise parallelizing complex algorithms (e.g. convolutional neural networks) (Chiroma *et al.*, 2019).

We developed ‘multi-omic integration with sparse value decomposition’ (MOSS) to handle these limitations. MOSS is a free and open-source R package that performs data integration and feature selection on large datasets. It combines the flexibility of sparse value decomposition (SVD) with parallel and in-disk computations to accommodate data sizes reaching biobank dimensions.

## 2 Implementation

The package’s primary function is called moss. Omic data are given to moss as a list where each element corresponds to a different omic (see help pages for function moss). Each omic enters the function as a numeric array. The rows of each array represent samples (e.g. a subject per row) and the column of each array an omic feature (e.g. expression of a gene). The rows of the different numeric arrays on the list need to be sorted in the same order (i.e. each row belongs to the same sample across omic blocks). Integration of omic blocks occurs by appending them, column-wise, into an extended matrix. Before making the extended matrix, blocks are normalized and standardized. If missing values are present, they are imputed by the mean. The effects of potential confounders can be internally adjusted by giving moss a data frame, vector or matrix with covariates. When omic blocks are too big to be handled in memory, File-backed Big Matrix (FBM) (Privé *et al.*, 2018) can be passed to moss. For this task, the package bigstatsr (Privé *et al.*, 2018) must be installed. Suppose the omic blocks fit in memory but are still too large to be handled in a reasonable time. In that case, moss allows turning the omic blocks into FBM objects internally.

MOSS performs a sparse singular value decomposition (sSVD) on the integrated omic blocks to obtain latent dimensions as sparse factors (i.e. with zeroed out elements), representing variability across subjects and features. Sparsity is imposed via Elastic Net (Zou *et al.*, 2005) (EN) on the sSVD solutions. MOSS allows an automatic tuning of the number of elements different from zero, adapting the procedure in Shen and Huang (2008). The primary output of MOSS is a list with the results of standard (dense) and sSVD. However, a flexible set of arguments extends the output to include cluster analysis, non-linear embedding and accompanying visualizations (Supplementary Information). Further statistical and algorithmic details and a description of moss’ arguments, plus examples of usage, are provided in Supplementary Information.

## 3 Moss identifies informative omic features as competently as existing methods

**Fig. 1.**
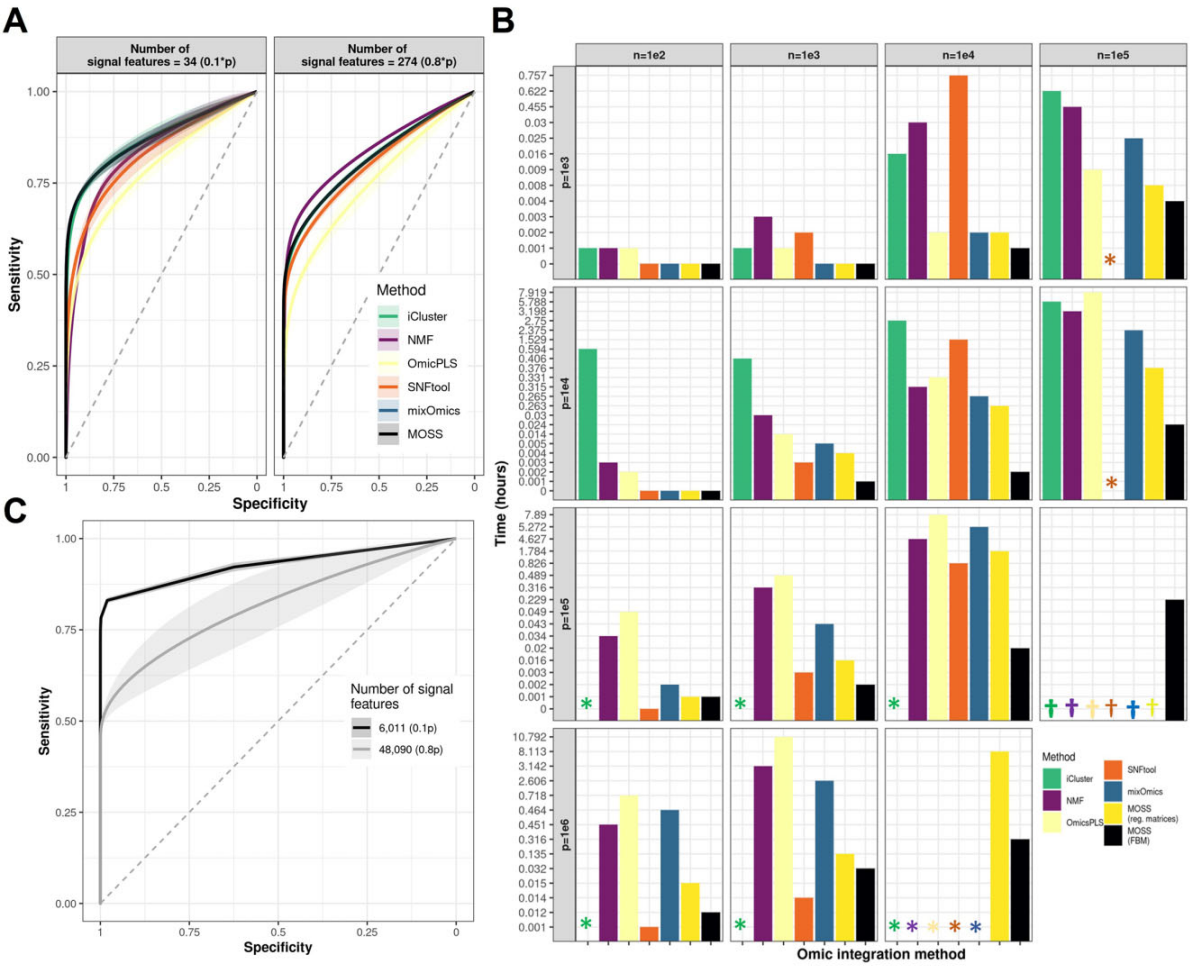
(**A**) Performance of MOSS and existing omic integration and features selection methods. Each panel represents a different proportion of informative features. Each curve represents the average specificity and sensitivity of features selection across 1000 random simulations for increasing sparsity degrees (e.g. null effect features). Confidence bands represent inter-simulations noise. (**B**) Comparison of computational time between MOSS and other methods. The plot shows the computational time taken by MOSS and five other omic integration methods. Scenarios corresponded to a different combination of samples (*n*) and features (*p*) in simulated data. Column panels represent the number of samples, and row panels represent the number of features. Each bar represents a different omic integration method. The y-axis shows the time in hours. The symbols ‘*’ and ‘†’ represent a method running for more than a day or crashing, respectively. MOSS was used with dense matrices (reg. matrices) or filed-backed big matrices (FBM). (**C**) Performance of MOSS on real high-dimensional data. The plot shows the performance of MOSS on simulations using data presented in (González-Reymúndez and Vázquez (2020). Different colors represent alternative proportions of features with signals

MOSS matches the performance of current analogous methods (Fig. 1A). To illustrate this point, we compared MOSS against existing methods of omic integration and feature selection. This comparison was done in terms of the methods ability to detect informative features. The methods included iCluster (Shen *et al.*, 2009), NMF (Gaujoux and Seoighe, 2010), SNFtool (Wang *et al.*, 2014), mixOmics (Rohart *et al.*, 2017) and OmicsPLS (el Bouhaddani *et al.*, 2018). The data consisted of simulations on top of gene and protein expression profiles from breast tumors from The Cancer Genome Atlas (TCGA; Chang *et al.*, 2013) repository (see Supplementary Information) and supplied within mixOmics. In each simulation, omic features were decorrelated by randomly shuffling tumors, one feature at a time. To define informative features in each simulation, a subgroup of randomly chosen features was left intact. These features conserved the naturally occurring correlation present in the data. The two scenarios compared used 10% and 80% of the total features to define the signal. A total of 1000 random simulations were run by scenario. Figure 1A shows MOSS’s ranking amongst the best performance methods. When using strict variable selection (EN parameter equal to 1), MOSS’s performance is inversely related to the number of informative features. In scenarios with a larger number of informative features, methods like NMF, more suitable for dense solutions, are more sensible. However, MOSS can compensate for the loss in sensitivity by compromising variables selection in favor of shrinkage (e.g. by setting EN parameter to values between 0 and 1).

## 4 Moss requires less computational time than existing methods and scales to datasets reaching biobank sizes

One of MOSS's essential capabilities is the handling of big data. While other tools demonstrate similar analytical performance (Fig. 1A), MOSS is specifically designed for big data. As a result, even when regular R matrices are used (i.e. omic data handled in RAM), MOSS can still perform in a short amount of time compared to other omic integration and feature selection methods (Fig. 1B). For huge datasets (e.g. scenario *n* = 1e5 and *p* = 1e6 in Fig. 1B), tuning of degree of sparsity with MOSS becomes prohibitive. However, dense solutions are still possible (i.e. without imposing sparsity).

## 5 Moss can be applied to high-dimensional real datasets

In González-Reymúndez and Vázquez (2020), we showed that MOSS could also retrieve biologically meaningful results from real data. Figure 1C shows the results of applying the above simulation scheme to data used in González-Reymúndez and Vázquez (2020), consisting of ∼60 000 features from whole-genome gene expression profiles, DNA methylation and copy numbers across ∼5000 tumors from 33 different cancer types.

## 6 Conclusions

Omic integration emerged as a group of techniques to collectively analyze multiple omic data layers and retrieve helpful information of shared biological processes (Hasin *et al.*, 2017). However, the computational and statistical tools used to carry out these tasks are constantly challenged by the vast amount of data generated (Conesa and Beck, 2019; Gomez-Cabrero *et al.*, 2014). As a result, omic integration can become a vast and challenging problem. Consequently, existing algorithms can become painfully slow or impossible to run.

As a features selection tool, MOSS performance is best as the number of signal features decreases (e.g. some signaling pathways affected in cancer, such as canonical MAPK pathway; Braicu *et al.*, 2019). However, lower performance for a larger number of signal features is an unsolved challenge among omic integration and feature selection methods (Tini *et al.*, 2017). In MOSS, this performance could be increased by compromising variable selection in favor of shrinking by varying the value of the EN parameter. For instance, in González-Reymúndez and Vázquez (2020), a EN parameter value of 0.5 was used to show MOSS's ability to detect clusters of tumors beyond original diagnoses and molecular signatures of potential therapeutic use. The training of this additional parameter, however, can drastically increase computational time, particularly for large datasets. More sophisticated alternatives might involve the use different penalties by omic block or set of features, a capability that we are considering for future versions of MOSS.

Despite its benefits as a data integration and mining tool, MOSS lacks statistical inference to support feature selection. Future versions of MOSS can deal with these limitations by adopting fast bootstrap techniques applied to high-dimensional SVD (Fisher *et al.*, 2016). In addition to unsupervised analysis, MOSS can fit supervised analyses via partial least squares, linear discriminant analysis and low-rank regressions. Nevertheless, these options are currently limited by the lack of cross-validation schemes to evaluate supervised models and address their performance.

In sum, MOSS is a flexible and fast tool to perform data integration. It shares capabilities with popular methods, including estimation of latent data dimensions, feature selection and convenient graphical displays. Nevertheless, unlike these methods, MOSS integrates datasets too large to be handled in RAM and requires considerably shorter amounts of time.
